# Cure of Chronic Viral Infection and Virus-Induced Type 1 Diabetes by Neutralizing Antibodies

**DOI:** 10.1080/17402520600800721

**Published:** 2006

**Authors:** Mette Ejrnaes, Matthias G. von Herrath, Urs Christen

**Affiliations:** 1 La Jolla Institute for Allergy and Immunology San Diego CA USA; 2 Johann Wolfgang Goethe University Frankfurt am Main Germany

## Abstract

The use of neutralizing antibodies is one of the most successful methods to interfere with receptor-ligand interactions *in vivo*. In particular blockade of soluble inflammatory mediators or their corresponding cellular receptors was proven an effective way to regulate inflammation and/or prevent its negative consequences. However, one problem that comes along with an effective neutralization of inflammatory mediators is the general systemic immunomodulatory effect. It is therefore important to design a treatment regimen in a way to strike at the right place and at the right time in order to achieve maximal effects with minimal duration of immunosuppression or hyperactivation. In this review we reflect on two examples of how short time administration of such neutralizing antibodies can block two distinct inflammatory consequences of viral infection. First, we review recent findings that blockade of IL-10/IL-10R interaction can resolve chronic viral infection and second, we reflect on how neutralization of the chemokine CXCL10 can abrogate virus-induced type 1 diabetes.


*Clinical & Developmental Immunology*, June–December 2006; 13(2–4): 337–347

Taylor & Francis

Taylor & Francis Group

### Cure of chronic viral infection and virus-induced type 1 diabetes by neutralizing antibodies

METTE EJRNAES[1], MATTHIAS G. VON HERRATH[1], & URS CHRISTEN[1,2,†]

[1]*La Jolla Institute for Allergy and Immunology, San Diego, CA, USA, and* [2]*Johann Wolfgang Goethe University, Frankfurt am Main, Germany*

**Abstract**

The use of neutralizing antibodies is one of the most successful methods to interfere with receptor-ligand interactions *in vivo*. In particular blockade of soluble inflammatory mediators or their corresponding cellular receptors was proven an effective way to regulate inflammation and/or prevent its negative consequences. However, one problem that comes along with an effective neutralization of inflammatory mediators is the general systemic immunomodulatory effect. It is therefore important to design a treatment regimen in a way to strike at the right place and at the right time in order to achieve maximal effects with minimal duration of immunosuppression or hyperactivation. In this review we reflect on two examples of how short time administration of such neutralizing antibodies can block two distinct inflammatory consequences of viral infection. First, we review recent findings that blockade of IL-10/IL-10R interaction can resolve chronic viral infection and second, we reflect on how neutralization of the chemokine CXCL10 can abrogate virus-induced type 1 diabetes.

**Keywords:** *Chronic infection, LCMV, autoimmune disease, dendritic cells, CXCR3, molecular mimicry*

### Introduction

Lymphocytic choriomeningitis virus (LCMV) infection of mice has proven to be one of the most informative experimental systems for investigating various aspects of virology and immunology. The various LCMV models offer several acute and persistent infection systems that are well suited to reflect immune kinetics as they might occur in a variety of human chronic infections. In addition, transgenic expression of LCMV proteins as model target antigens in the pancreas or the CNS is being used in animal models for type 1 diabetes (T1D) (Ohashi et al. 1991; Oldstone et al. 1991) and multiple sclerosis (MS) (Evans et al. 1996), respectively.

LCMV belongs to the arena virus family and is a natural pathogen for both humans and mice (Zinkernagel and Doherty 1974). The viral genome is comprised of two single stranded RNA segments; both segments are antisense, each encoding two proteins. The larger (L) segment encodes the L protein (the viral polymerase) and the Z protein with an undefined function. The short (S) segment encodes the nucleoprotein (NP) and the glycoprotein (GP), which undergoes post-translational cleavage to generate the two mature GPs, GP1 and GP2 (Borrow and Oldstone 1997). LCMV can cause acute or persistent infection *in vivo* depending on the strain, route of infection and dose. Whereas adult mice inoculated intravenously with LCMV strain Armstrong (LCMV-Arm) rapidly clear the infection and remain immune-competent with the establishment of a stable memory T cell pool (Figure 1) (Marker and Volkert 1973; Moskophidis et al. 1987), inoculation with the LCMV variant Cl13, which differs from its parent (LCMV-Arm) virus at only one amino acid positions in the virus GP is associated establishment of a chronic infection. This chronic infection is associated with both the functional impairment and deletion of virus-specific CD8 T cells

Correspondence: M. Ejrnaes, Department of Developmental Immunology, La Jolla Institute for Allergy and Immunology, 10355 Science Center Dr, San Diego, CA 92121, USA. Tel: 1 858 558 3581. Fax: 1 858 558 3579. E-mail: mejrnaes@liai.org
†Tel: 49 69 6301 83105. Fax: 49 69 6301 7942. E-mail: christen@med.uni-frankfurt.de

ISSN 1740-2522 print/ISSN 1740-2530 online © 2006 Taylor & Francis
DOI: 10.1080/17402520600800721

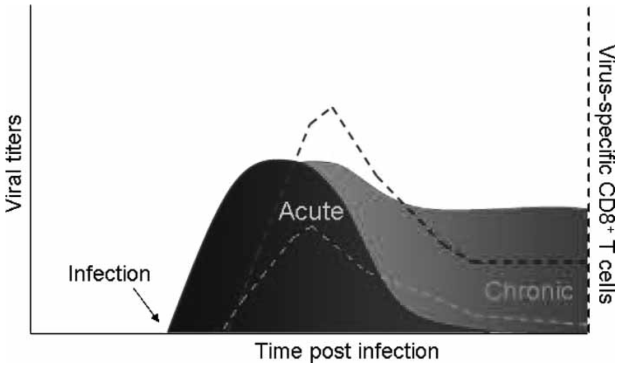

Figure 1. Mice infected with medium dose LCMV Armstrong develop an acute viral infection characterized by an initial expansion of anti-viral specific T CD8 + cells (blue line) with the ability to efficiently eliminate virus infected target. During their expansion, viral antigen-specific $CD8^{+}$T cells acquire effector functions, including the ability to rapidly produce cytokines. Viral clearance is dependent on the presence of $CD8^{+}$ virus-specific CTLs (Zinkernagel and Welsh 1976; Oldstone et al. 1986; Jamieson et al. 1987; Moskophidis et al. 1987); and the infection is cleared by day 10–12 from blood and spleen (Blue shaded area). After expansion of effectors and their contraction, a stable memory $CD8^{+}$T cell population is generated that can be maintained in the absence of antigen (blue line) (Lau et al. 1994; Murali-Krishna et al. 1999; Homann et al. 2001; Kaech et al. 2002). In contrast, infection with Cl13, results in a prolonged infection that persists (red shaded area). This chronic infection is associated with both the functional impairment, deletion of virus-specific CD8 T cells and due to lack of establishment of a memory T cells pool inability mount sufficient recall responses (red line).

and general immunosuppression (Moskophidis et al. 1993; Lin and Welsh 1998).

### Chronic-persisting protracted LCMV variant Clone 13

The LCMV variant Clone 13 (Cl13) was originally isolated from the spleen of a 2 month old mouse infected at birth with LCMV-Arm (Matloubian et al. 1990). The molecular basis of persistence and suppression of the anti-LCMV cytotoxic T lymphocyte (CTL) response has been mapped to a single amino acid change in LCMV-GP; LCMV-Arm has a phenylalanine at amino acid position 260, whereas the variant Cl13 has a leucine at this position (Salvato et al. 1991; Dockter et al. 1996). Infection of mice with high doses of Cl13 leads to a persistent infection that is associated with a lack of viral antigen specific CD8 effectors (Figure 1) (Moskophidis et al. 1993; Lin and Welsh 1998).

A peripheral membrane protein, alpha-dystroglycan ($\alpha$-DG), was identified as the receptor for LCMV (Cao et al. 1998). Additionally, it was observed that Cl13 binds more strongly to $\alpha$-DG as compared to the Armstrong strain (Smelt et al. 2001). The affinity of the viral binding to its receptor might be a crucial element in determining the outcome of infection with LCMV Armstrong (lower affinity to $\alpha$-DG) versus Cl13 (high affinity to $\alpha$-DG). It was shown that differences in binding affinities of LCMV strains to $\alpha$-DG correlated with viral tropism and disease kinetics (Smelt et al. 2001). LCMV-Arm and Cl13 appear to exhibit differences in their tropism within the spleen, with Cl13 causing a higher level of infection of antigen presenting cells (APCs) in the white pulp, including periarterial interdigitating DCs. Cl13 could thereby render these cells targets for more effective destruction by the antiviral $CD8^{+}$ CTL response which is induced at an early time point following infection with Cl13 compared to LCMV-Arm. As a consequence this CD8-dependent destruction of dendritic cells (DCs) in the spleen of Cl13-infected mice leads to the ensuing immunosuppression (Borrow et al. 1995). *In vitro* studies showing a decline of DCs after infection with Cl13 but not LCMV-Arm support the notion that the loss was related to infection (Sevilla et al. 2004). Additionally, it was reported that DCs obtained from cultures infected with Cl13, but not LCMV-Arm were non-functional, as demonstrated by their ability to stimulate allogeneic T cells (Sevilla et al. 2004). Interestingly, upon closer look, this publication also shows that the loss of DCs was mostly restricted to the CD11c + $CD8\alpha^{+}$ population and affected CD11c + $CD8\alpha^{neg}$ DCs to a much lesser extent.

### IL-10 as a modulator of APC function

Interleukin-10 (IL-10) is an immunosuppressive cytokine with implications for various immune and inflammatory diseases. It inhibits a broad spectrum of cellular immune responses, acting on APCs by, i.e. preventing DC maturation and thereby keeps the cells in an immature state and T cells by inhibiting pro-inflammatory cytokine production. Investigations have revealed that IL-10 play important roles in blocking cytokines production (Fiorentino et al. 1991) costimulatory molecules expression, there under MHC class II, CD80 and CD86 (Ding et al. 1993; Willems et al. 1994) as well as chemokine secretion (Jinquan et al. 1993; Kasama et al. 1994) and modification of chemokine receptor expression (Sozzani et al. 1998; Takayama et al. 2001) (for review see Pestka et al. 2004). Apart from the reported potent immune-modulatory effects on APCs, IL-10 also affects $T_H1$- and $T_H2$-type immune responses, as evidenced by findings in asthma, transplantation and autoimmunity models (Moore et al. 2001). IL-10 regulates the proliferation and differentiation of $T_H1$-type T lymphocytes, which appear to control many effector immune responses (i.e. host defense, anti-tumor immunity, autoimmunity) *in vivo* (Fiorentino et al. 1991). In different cell types, the duration of stimulation affects IL-10 expression differentially. In T cells IL-10 expression occurs shortly after stimulation and the levels of IL-10

increase with the duration of stimulation (Wolk et al. 2002). Elevated levels of IL-10 mRNA have been observed in immune-responsive versus non-responsive metastatic melanoma lesions (Mocellin et al. 2001). Moreover, treatment with a combination of anti-IL-10 receptor (IL-10R) monoclonal antibody (mAb) and toll-like receptor 9 (TLR9) ligands has been shown to have potent therapeutic anti-tumor effects (Vicari et al. 2002; Vicari and Trinchieri 2004), pointing to the role of IL-10 in the development of cancer. Furthermore, IL-10 has also been shown to play a role in the establishment of certain chronic viral infections such as human immunodeficiency virus (HIV-1) (Granelli-Piperno et al. 2004), hepatitis C (HCV) and human cytomegalovirus (HCMV) infections (Rigopoulou et al. 2005). Interestingly, an increase in systemic IL-10 production has been demonstrated upon HCV infection (Accapezzato et al. 2004), and in some cases of infection with HIV (Akridge et al. 1994; Ameglio et al. 1994; Autran et al. 1995; Granelli-Piperno et al. 2004; Ji et al. 2005).

### Current immunotherapeutic approaches in persistent infections

As the hallmark of chronic infection is an impaired virus specific effector cell response, numerous efforts have been undertaken to increase anti-LCMV immunity. Conventional immunotherapy attempting to augment anti-viral immunity directly in persistent infected individuals has failed to affect the outcome so far, but lowering the viral antigenic load (by interferons or anti-viral drugs) has clear beneficial effects. Additionally, specific immunization strategies have been combined with direct anti-viral drug treatments, for example protease inhibitors and highly active antiretroviral therapy (HAART) in HIV (Palella et al. 1998) and interferon administration, and ribavirin in hepatitis infections (Torriani et al. 2004; Kamar et al. 2005). In all of the situations where anti-viral drugs were employed, viral loads were significantly reduced and, for HIV, long-term deleterious consequences of the persistent infection were decreased. Interestingly, the outcome of ribavirin administration in chronic HCV was not quite as promising, although viral titers were lowered, since liver fibrosis was enhanced, possibly as a rather direct effect of the drug (Torriani et al. 2004; Kamar et al. 2005). However, complete elimination of the pathogen has remained elusive, despite the fact that anti-viral immunity was significantly increased in many instances.

Much attention has been paid to the role if IL-10 as a potent anti-inflammatory immuno-suppressive cytokine with important potential clinical applications. So far it remains unclear, whether IL-10 affects the outcome of infection, amount of immunopathology and complications and could be the actual cause for persistence. One major concern in clinically manipulating the levels of IL-10 is its critical role in the immune homeostasis. Long-term application of IL-10 could cause immunodeficiency, whereas continuous use of anti-IL-10 may lead to hyper immune reactivation. However, blocking the cytokine receptor itself for a short period of time is a relative novel approach to control the signaling effects of the cytokine. To gain further insight into the function of IL-10 in the establishment and maintenance of persistent viral infections, we have investigated how blockade of the IL-10/IL-10 receptor signaling pathway affects LCMV chronic infection in its natural host, the mouse.

### Neutralization of IL-10 resolves chronic viral infection

LCMV Cl13-infection results in a prolonged period of elevated IL-10 production, which is predominantly produced by CD4 + T cells already early upon infection (Ejrnaes et al. 2006). This interestingly coincides with the loss of the systemic CTL response against the virus. Treatment with a neutralizing IL-10R antibody that was previously shown to block IL-10/IL-10R interaction *in vitro* (BD Biosciences) results in accelerated viral clearance (Ejrnaes et al. 2006). This is associated with a numeric increase of total spleen cells in comparison to non-treated mice indicating that development of lymphopenia is reversed upon anti-IL10R mAb treatment (Ejrnaes et al. 2006). Furthermore, this rapid resolution of viral infection is associated with diminished levels of endogenous IL-10 and enhanced anti-viral $CD8^+$ memory T cell responses (Ejrnaes et al. 2006). Importantly, overall clinical appearance is improved through such an intervention as reflected in an increase in bodyweight, healthy shiny coat, and increase in physical activity (Ejrnaes et al. 2006). Interestingly, this protection from chronic infection was achieved with only a few injections of neutralizing anti-IL-10R mAb immediately after virus infection. Thus, the duration of treatment could be minimized in order to avoid long-term systemic immunosuppressive effects.

Previous reports suggest that different DC subsets vary in their ability to prime effector T cells (Liu 2001), and in particular, evidence suggests that DCs can be converted to APCs, which skew the immune response towards $T_H2$-domination, when treated with anti-inflammatory cytokines such as IL-10 (Buelens et al. 1995; Liu 2001). In this context we found that activation of LCMV-specific T cells in chronically infected mice is preferentially achieved by $CD8\alpha^-$ DCs, which induced IL-10 production by virus-specific $CD4^+$T cells (Ejrnaes et al. 2006). Subclasses of DCs have been shown to have the potential to differentially skew cytokine production towards

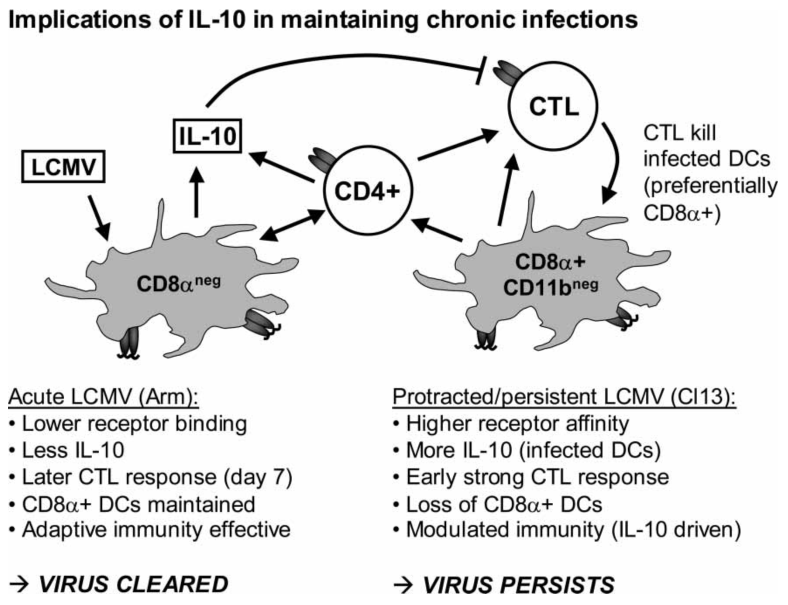

Figure 2. LCMV Cl13 persistence is the result of an early CTL-mediated loss of $CD8\alpha^+$ DCs, which indirectly supports immune responses high in IL-10 production that might be predominantly driven by the remaining $CD8\alpha^{neg}$ $CD11c^+$ DCs.

$T_H1$- or $T_H2$-profiles (Mosmann and Coffman 1989). Notably, it has been suggested that $CD8\alpha^-$ DCs induce $T_H2$-profiles whereas $CD8\alpha^+$ DCs preferentially stimulate IFN-γ production and therefore induce $T_H1$ profiles (Maldonado-Lopez et al. 1999), a result we could confirm when analyzing the ability of $CD8\alpha^+$ and $CD8\alpha^-$ DCs to polarize naïve LCMV-reactive $CD4^+$T cells (Ejrnaes et al. 2006). The generalized state of lymphopenia induced by Cl13 infection might be mediated by APCs inducing IL-10 production. However, the mechanisms by which IL-10 enables Cl13 to persist are unknown. IL-10 could either down-regulate pro-inflammatory responses in a general manner or, more specifically, inhibit the induction or expansion of anti-viral CTLs. In fact, IL-10 may directly decrease the viability of $CD8\alpha^+$ DCs as has been previously suggested (Maldonado-Lopez et al. 2001; Re and Strominger 2004). In the state of chronic Cl13 infection, prominent $CD8\alpha^-$ DCs with reduced ability to prime $T_H1/T_C1$ effectors "by default" become the modulators of the T cell response and thus derail anti-LCMV immunity through the production of IL-10 (Figure 2). While the precise mode of action of IL-10 is unknown, LCMV-specific $CD4^+$T-cells activated in this context may in turn acquire the ability to produce IL-10 and provide inappropriate or insufficient anti-viral help to other cell types, in particular $CD8^+$T cells, thus leading to persistent infection (Figure 2). Additionally, it is possible that the remaining $CD8\alpha^-$ DCs, which appear ill-equipped to propagate anti-viral effectors, will continue to support IL-10 production. The resulting high concentration of IL-10 in the milieu may thus lead to further modulation of DC function and in this way become a self-fulfilling phenomenon (Figure 2). As a consequence, only disruption of IL-10 signaling will have the ability to break this vicious circle and enable the recovery of appropriate anti-viral immunity by the infected host. Since blockade of IL-10 signaling likely directly acts on DCs, a central switch in immunity could be implanted in this way directly at the core, where most immune responses are orchestrated.

### Virus-induced type 1 diabetes

Another possible consequence of virus infection and its accompanying inflammatory cascades is the initiation or acceleration of autoimmune disease. One scenario that might be involved in the etiology of autoimmune disease is that virus infection could break self-tolerance due to an inherent structural similarity to self-components (Oldstone 1989; Cantor 2000; Olson et al. 2001; Miller et al. 2001; Christen and Herrath 2004; Christen et al. 2004b). This concept of molecular mimicry has been integrated into animal models for various human autoimmune diseases such as T1D, MS, or systemic lupus erythematosus (SLE) (for review see Christen and von Herrath 2004a). The RIP-LCMV mouse model for T1D that was established in 1991 in the laboratories of Michael Oldstone (Oldstone et al. 1991) and Rolf Zinkernagel (Ohashi et al. 1991). These mice express the GP or NP of LCMV-Arm in the insulin-producing beta-cells of the islets of Langerhans in the pancreas. Such beta-cells are the target of the auto-aggressive immune system in T1D. Since the transgenically expressed viral protein is considered a component of "self", RIP-LCMV mice

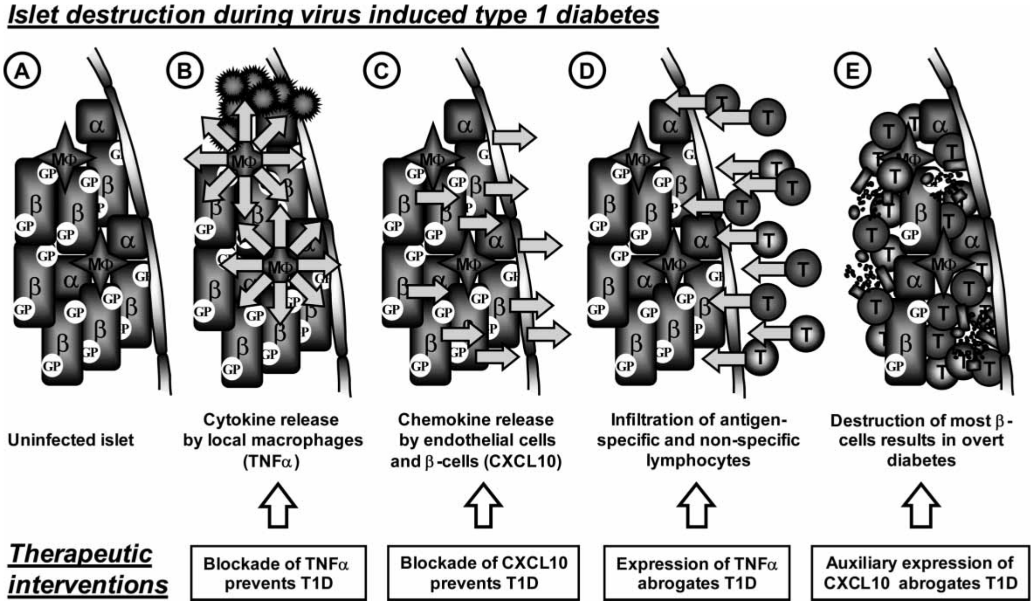

Figure 3. Therapeutic intervention during the ongoing destruction of islet cells in the pancreas.—After LCMV infection active virus is found in the pancreas but not necessarily in the islets. In an initial stage local resident macrophages or dendritic cells are activated and release pro-inflammatory cytokines, such as TNFα. Blocking of TNFα at this early stage after infection can abrogate T1D (Christen et al. 2001; Christen and von Herrath 2004c) (panel B). Chemokines, most prominently CXCL10, are released by endothelial cells and β-cells (Frigerio et al. 2002; Christen et al. 2003) (panel C) which in turn attract islet-specific auto-aggressive T lymphocytes as well as non-specific bystander cells into the islets of Langerhans (Christen et al. 2003; Rhode et al. 2005) (panel D). At that time neutralization of CXCL10 prevents the subsequent development of T1D by interfering with the migration of auto-aggressive T lymphocytes to the pancreas (Christen et al. 2003). When not prevented from doing so, the first islet-specific auto-aggressive CD8 cells destroy some β-cells by perforin dependent cytolysis resulting in release of β-cell antigens (panel E). These antigens include transgenic viral proteins and additional non-viral components and are processed and presented to infiltrated CD8 and CD4 cells by APC (panel E). In the terminal stage of immunopathogenesis the majority of β-cells are being destroyed by auto-aggressive CD8 cells in a IFNγ dependent manner (Seewaldt et al. 2000). It is only in this final stage where clinically overt diabetes is apparent and can be assessed by blood glucose measurements. The destructive process at this end stage can however be reversed in the presence of high local concentrations of TNFα (islet-specific TNFα expression in a transgenic system) which can induce a status of hyper-activation resulting in apoptosis of auto-aggressive CD8 (Christen et al. 2001; Christen and Von Herrath 2002) (panels D and E). Similarly, expression of CXCL10 at an auxiliary site, such as the PDLN, can cause a recruitment of auto-aggressive CD8 cells away from the islets and a subsequent apoptosis in the PDLN by hyper-activation (Rhode et al. 2005).

are tolerant and do not mount an immune response to the LCMV protein. However, infection with LCMV-Arm itself breaks this self-tolerance, auto-aggressive LCMV-specific T cells are generated and expanded, the beta-cells are being destroyed and ultimately clinical overt diabetes results (Figure 3). In the context of this review article it is important to reiterate here that LCMV-Arm is one of the isolated LCMV strain that has no immunosuppressive properties and does not result in persistent infection. In the past this model was used to investigate mechanisms of how autoimmune processes are involved in the pathogenesis of T1D and to evaluate possible treatments for human T1D in an animal model.

Just as proposed for human T1D, the onset of diabetes in RIP-LCMV mice depends on the action of both, autoreactive CD4 and CD8 T-cells and correlates with the numbers of auto-aggressive lymphocytes generated. In accordance, the incidence of disease varied between the individual transgenic lines ranging from 2 weeks (RIP-GP lines) to 1–6 months (RIP-NP lines). Further studies revealed the mechanism involved in the rapid compared to the slow onset diabetes: Transgenic lines expressing the LCMV-GP transgene exclusively in the β-cells of the islets manifested rapid-onset T1D (10–14 days after viral challenge) (von Herrath et al. 1994). In these lines the high systemic numbers of auto-aggressive CD8 T-cells were sufficient to induce diabetes and did not require help from CD4 cells. In contrast, in lines expressing the LCMV-NP transgene in both the β-cells and in the thymus, T1D took longer to occur after subsequent LCMV challenge. Several lines of evidence indicated that in RIP-NP mice the anti-self (viral) CTL were of lower affinity and that CD4 T-cells were essential to generate anti-self (viral) CD8 lymphocyte-mediated T1D (von Herrath et al. 1994). In addition, mouse models in which transgene-encoded “target-antigens” are expressed in the pancreatic β-cells, such as the RIP-LCMV and the INS-HA (Lo et al. 1992; Lo et al. 1993) mouse, have demonstrated that the presence of auto-aggressive T-cells alone is not enough to cause disease. For example, when RIP-LCMV mice were crossed with mice expressing an inactive mutated form of IFNγR the diabetes incidence was drastically reduced (Seewaldt et al. 2000). These results indicate that unspecific “bystander factors”, such as cytokines and chemokines generated during the acute inflammation after LCMV infection, are important to drive the auto-aggressive response (β-cell destruction) in “antigen-specific” models for T1D.

Hence, the RIP-LCMV model has become a very useful tool to study the etiology and the mechanisms of human autoimmune diabetes and to evaluate possible treatments, such as blockade of specific inflammatory factors, as discussed below (Christen et al. 2001; Christen et al. 2003), oral tolerance induction (Homann et al. 1999) or DNA-vaccination (Coon et al. 1999). Besides having a clearly defined initiation point (LCMV-infection), the advantage of the RIP-LCMV system over other established models for T1D, such as the NOD mouse, is the presence of extensively characterized target antigens (GP, NP). The immune response against these target antigens can be visualized using flow cytometry by stimulation of splenocytes with LCMV-peptides or direct staining of CD8 T-cells with MHC class (-peptide tetramers (Murali-Krishna et al. 1998). In addition, we recently demonstrated tracking of LCMV-specific CD8 T-cells by *in situ* MHC class (-peptide staining of quick-frozen tissue sections (McGavern et al. 2002).

### CXCL10—a sentinel chemokine during inflammation

Among the inflammatory mediators generated upon LCMV-infection of the pancreas, CXCL10 proved to play a key role in imprinting a pattern for the subsequent development of autoimmune disease in the RIP-LCMV model. In general, ligands of the CXCR3 chemokine receptor including CXCL10 (interferon-induced protein of 10 kDa, IP-10), CXCL9 (monokine induced by gamma-interferon, Mig), and CXCL11 (interferon-inducible T-cell α chemoattractant, I-TAC) have been suggested to play an essential role during inflammation and autoimmunity. CXCR3 chemokine ligands are mainly expressed by keratinocytes, macrophages, fibroblasts, and endothelial cells upon stimulation with IFNγ or TNFα (Luster et al. 1985; Luster and Ravetch 1987) but are also generated by activated T-cell hybridomas, normal T-cells, and thymocytes (Gattass et al. 1994). CXCL10 was suggested to function as a “sentinel molecule” in host defense against viruses (Liu et al. 2000) and is generated very early after infection with a wide variety of viruses such as HIV, adenovirus, LCMV, Theiler’s virus and mouse hepatitis virus (Asensio and Campbell 1997; Lane et al. 1998; Charles et al. 1999; Hoffman et al. 1999; Kolb et al. 1999). Further, CXCR3 chemokine ligands are have been implicated in the host defense against foreign pathogens by promoting local inflammation. Blockade of CXCL10 disturbed the control of parasite propagation after *Toxoplasma gondii* infection by abrogating T-cell migration into tissues and impairing antigen-specific T-cell function resulting in amplified tissue parasite burden and an increased mortality (Khan et al. 2000). Further, transgenic (tg) CXCL10 expression in keratinocytes resulted in delayed wound healing and disorganized neo-vascularization due to a more intense inflammatory phase (Luster et al. 1998).

CXCR3 is the only cellular receptor for CXCL9, CXCL10 and CXCL11 identified to date, and is predominantly found on activated Th1-type T-cells (Loetscher et al. 1996; Sallusto et al. 1998). Thus, CXCR3 chemokines direct the anti-viral defense towards the more aggressive type 1 T-cell-domination and act as “bystander effectors” that unspecifically activate T-cells (including autoreactive T-cells) and subsequently drive an auto-aggressive immune response that may result in autoimmune disease.

### Unique role of CXCL10 in imprinting a pattern for autoimmune disease

Data from our lab suggest that, among CXCR3 chemokines, CXCL10 plays a unique role in imprinting a pattern for the subsequent development of autoimmunity (Christen et al. 2003; Christen and von Herrath 2004b). As previously reported for infection of the CNS by various viruses (Asensio and Campbell 1997; Lane et al. 1998; Charles et al. 1999; Hoffman et al. 1999; Kolb et al. 1999), infection of mice with LCMV caused a very rapid and strong expression of CXCL10 in the pancreas (Christen et al. 2003). Interestingly, CXCL10 was the only chemokine whose expression was strongly induced as early as 1 day after infection. The other two CXCR3 chemokine ligands were either expressed later (CXCL9) or were only faintly upregulated (CXCL11). Further, chemokines such as CCL3 (MIP-1α) or CCL5 (RANTES) were not increased until day 7 post-infection, a time where most pro-inflammatory cytokines including TNFα, and IFNγ are strongly expressed as well (Christen et al. 2003). Very recently, the important role of CXCL10 during virus-induced diabetes was underlined in RIP-CXCL10 transgenic mice, which showed a spontaneous infiltration of islets by a mixed leukocyte population (Rhode et al. 2005). These mice were not diabetic but had a reduced capacity to response to a high glucose challenge possibly due to “inflammatory stress” to the islets (Rhode et al. 2005). In addition, when crossed to the RIP-LCMV mouse line the RIP-CXCL10 mice had accelerated T1D after infection with LCMV (Rhode et al. 2005). Thus, CXCL10 is a prime candidate for a neutralization attempt to rescue mice from autoimmune disease.

### Neutralization of CXCL10 abrogates type 1 diabetes

We used a very characterized neutralizing monoclonal antibody to CXCL10 that was previously shown to block the biological activity of CXCL10 *Toxoplasma gondii* infection model (Khan et al. 2000). Briefly, neutralization of CXCL10 inhibited the influx of activated T-cells into tissue and a 1000-fold higher parasite burden resulting in a higher mortality of infected mice (Khan et al. 2000). In our T1D mouse model we found that indeed blockade of CXCL10 with this neutralizing antibody abrogated disease in $>60\%$ of all LCMV-infected mice (Christen et al. 2003). In contrast blockade of CXCL9 with a neutralizing anti-CXCL9 antibody neither reduced the incidence nor prolonged the onset of diabetes (Christen et al. 2003). Mechanistically, blockade of CXCL10 interfered with the expansion of LCMV-specific auto-aggressive CD8 T-cells and their migration to the pancreatic islets of Langerhans (Christen et al. 2003). Thus, neutralization of one critical inflammatory factor at the right time could prevent the subsequent development of autoimmune disease. It is important here to note, that the treatment had to be administered at the precise time when CXCL10 was expressed at high density. Rescued mice received five intravenous injections of 100 μg neutralizing antibody at days 0, 1, 2, 4 and 6 post infection. Treatment of mice before LCMV-infection or at a later time (days 7–14) was not successful in preventing autoimmune disease (U.CH. unpublished observations and reference (Christen et al. 2003)).

The same monoclonal anti-CXCL10 antibody was used to neutralize CXCL10 in the CNS of mice with experimental autoimmune encephalomyelitis (EAE) (Fife et al. 2001). Similar to our experiences in the RIP-LCMV model, CXCL10 neutralization decreased the accumulation of antigen-specific lymphocytes to the site of autoimmune damage (Fife et al. 2001). A different approach to neutralize CXCL10 was recently published by the Narumi group who made use of the non-obese diabetic (NOD) mouse model for spontaneous T1D (Morimoto et al. 2004). They administrated young NOD mice with a DNA plasmid encoding CXCL10. This DNA vaccination approach resulted in the *in vivo* generation of neutralizing anti-CXCL10 antibodies and suppressed the development of diabetes (Morimoto et al. 2004). In contrast to the findings in the RIP-LCMV model, the treatment did neither reduce insulitis nor alter the islet-specific immune response. It rather enhanced the proliferation of the insulin-producing β-cells resulting in a higher β-cell mass (Morimoto et al. 2004). Another interpretation of these data would be that CXCL10 neutralization relieves the islets from "inflammatory stress". Thus, β-cells from DNA-vaccinated NOD mice might proliferate at a normal rate whereas β-cells from untreated NOD mice show reduced proliferation rates and therefore insufficient insulin production. However, the experiments by Morimoto et al demonstrate that neutralization of CXCL10 can be successful even in a spontaneous model such as the NOD mouse.

It was previously demonstrated that CXCL10 is predominantly expressed by β-cells upon LCMV-infection of the pancreas (Frigerio et al. 2002; Rhode et al. 2005). However, recent observations in our group show that diabetogenic T-cell clones produce CXCL10 as well (Ejrnaes et al. 2005). In this study, CD8 T-cell clones specific for the islet antigens insulin, glutamic acid decarboxylase (GAD) and LCMV-GP were analysed for expression of various chemokines and cytokines. Interestingly, there was a clear correlation between diabetogenicity and the expression of CXCL10 (Ejrnaes et al. 2005). Additional CXCL10-expression by auto-aggressive T-cells might lead to a perpetuated infiltration rate of the islets and subsequent acceleration of the destructive process.

### Auxiliary CXCL10 expression can abrogate autoimmune disease

Whereas expression of CXCL10 at the site of autoimmune damage clearly accelerates the immunopathogenic process and exacerbates disease (Rhode et al. 2005), auxiliary expression of CXCL10 can, under certain circumstances, abrogate the ongoing autoimmune destruction. In the late 1980ies it was observed that infection of NOD mice with LCMV can be used as a therapeutic intervention to prevent T1D (Oldstone 1988; Oldstone 1990). In the RIP-LCMV model T1D could be abrogated by a secondary infection with one particular strain of LCMV (strain Pasteur, LCMV-Past) but not with LCMV-Arm (strain Armstrong) that was used for the initial induction of disease (Christen et al. 2004a). The major difference between those two LCMV strains, that share all the immunologically relevant epitopes, is a striking discrepancy in viral growth in the pancreatic draining lymph node (PDLN). Secondary infection with LCMV-Past resulted in higher viral titers in the PDLN when compared to the pancreas and to infection with LCMV-Arm. Along with this augmented viral proliferation a differential expression of the inflammatory chemokine CXCL10 between PDLN and pancreas was observed (Christen et al. 2004a). Interestingly, a similar increase in PDLN-specific CXCL10 expression could be detected in NOD mice infected with LCMV-Past as well as LCMV-Arm (Christen et al. 2004a). In both LCMV-Past-infected NOD and RIP-LCMV mice a significant decrease in the infiltration of islets and an increase in cellular apoptosis in the PDLN was detected (Christen et al. 2004a). Apparently, auto-aggressive cells migrated away from the islets and got stuck in the PDLN due to the high CXCL10 concentration. Once arrived at the highly inflamed PDLN the cells were hyper-activated and died by apoptosis. Thus, in such a scenario, the LCMV-infected, highly inflamed auxiliary site may the act as a filter for auto-aggressive lymphocytes (Christen and von Herrath 2005).

### Summary

Neutralizing antibodies have been widely used to interfere with receptor-ligand interactions *in vivo*. Blockade of soluble inflammatory mediators and/or their cellular receptors is an highly effective way to down-regulate inflammation or prevent its negative consequences. In contrast to non-specific chemical immunomodulators or systemic treatment with cytokines, such as IFN-α, which act on a relatively broad range and thus can cause either unwanted severe side effects or general immunosuppression or -activation, neutralizing monoclonal antibodies act in a more specific way by preventing the binding of key mediators of inflammation to their cellular receptor. Nevertheless, long-term systemic administration of neutralizing antibodies inactivates the function of such mediators in their natural role in defense against harmful pathogens, such as viruses. It is therefore important to design a therapeutic regimen in way to reach maximal efficacy with a minimal side effects. This can only be achieved by a detailed knowledge of the mechanisms of action and the expression kinetics of such inflammatory mediators. In this review we highlighted two recent findings from our lab that demonstrate that short term neutralization of inflammatory mediators can abrogate or reverse adverse effects of chronic inflammation. First, blockade of IL-10/IL-10R interaction can resolve chronic viral infection. Mechanistically, IL-10 neutralization restores the anti-viral immune response that was derailed by viral infection of a specific subgroup of APCs, namely the CD8α- DCs. Second, short-term neutralization of CXCL10 after in virus infection prevented the progression from inflammation to autoimmune disease in a mouse model for T1D. In both examples, the precise timing and duration of administration of neutralizing antibodies was critical for success. Similarly, treatment of LCMV-infected RIP-LCMV mice with TNFR55-IgG1 fusion protein to neutralize TNFα was only successful when given early after infection (Christen et al. 2001). The finding that transgenic expression of TNFα early after infection enhanced the incidence of T1D, whereas late TNFα-expression reversed the auto-destructive process, suggested that TNFα has actually a dual role in T1D depending on the time of its expression (Christen et al. 2001). Thus, for applications of neutralizing antibodies as therapeutic agents one has to consider carefully on one hand the precise timing of treatment in order to achieved the desired protective effect and on the other hand the duration of the treatment to avoid permanent suppression or hyper--activation of the immune system.

### References

Accapezzato D, Francavilla V, Paroli M, Casciaro M, Chircu LV, Cividini A, Abrignani S, Mondelli MU, Barnaba V. 2004. Hepatic expansion of a virus-specific regulatory CD8(+) T cell population in chronic hepatitis C virus infection. J Clin Invest 113:963–972.

Akridge RE, Oyafuso LK, Reed SG. 1994. IL-10 is induced during HIV-1 infection and is capable of decreasing viral replication in human macrophages. J Immunol 153:5782–5789.

Ameglio F, Cordiali Fei P, Solmone M, Bonifati C, Prignano G, Giglio A, Caprilli F, Gentili G, Capobianchi MR. 1994. Serum IL-10 levels in HIV-positive subjects: Correlation with CDC stages. J Biol Regul Homeost Agents 8:48–52.

Asensio VC, Campbell IL. 1997. Chemokine gene expression in the brains of mice with lymphocytic choriomeningitis. J Virol 71:7832–7840.

Autran B, Legac E, Blanc C, Debre P. 1995. A Th0/Th2-like function of CD4 + CD7- T helper cells from normal donors and HIV-infected patients. J Immunol 154:1408–1417.

Borrow P, Evans CF, Oldstone MB. 1995. Virus-induced immunosuppression: Immune system-mediated destruction of virus-infected dendritic cells results in generalized immune suppression. J Virol 69:1059–1070.

Borrow P, Oldstone MBA. 1997. Lymphocytic choriomeningitis virus. Viral Pathogenesis :593–627.

Buelens C, Willems F, Delvaux A, Pierard G, Delville JP, Velu T, Goldman M. 1995. Interleukin-10 differentially regulates B7-1 (CD80) and B7-2 (CD86) expression on human peripheral blood dendritic cells. Eur J Immunol 25:2668–2672.

Cantor H. 2000. T-cell receptor crossreactivity and autoimmune disease. Adv Immunol 75:209–233.

Cao W, Henry MD, Borrow P, Yamada H, Elder JH, Ravkov EV, Nichol ST, Compans RW, Campbell KP, Oldstone MB. 1998. Identification of alpha-dystroglycan as a receptor for lymphocytic choriomeningitis virus and Lassa fever virus. Science 282:2079–2081.

Charles PC, Chen X, Horwitz MS, Brosnan CF. 1999. Differential chemokine induction by the mouse adenovirus type-1 in the central nervous system of susceptible and resistant strains of mice. J Neurovirol 5:55–64.

Christen U, Benke D, Wolfe T, Rodrigo E, Rhode A, Hughes AC, Oldstone MB, Von Herrath MG. 2004a. Cure of prediabetic mice by viral infections involves lymphocyte recruitment along an IP-10 gradient. J Clin Invest 113:74–84.

Christen U, Edelmann KH, McGavern DB, Wolfe T, Coon B, Teague MK, Miller SD, Oldstone MB, von Herrath MG. 2004b. A viral epitope that mimics a self antigen can accelerate but not initiate autoimmune diabetes. J Clin Invest 114:1290–1298.

Christen U, Herrath MG. 2004. Initiation of autoimmunity. Curr Opin Immunol 16:759–767.

Christen U, McGavern DB, Luster AD, von Herrath MG, Oldstone MB. 2003. Among CXCR3 chemokines, IFN-gamma-inducible protein of 10 kDa (CXC chemokine ligand (CXCL) 10) but not monokine induced by IFN-gamma (CXCL9) imprints a pattern for the subsequent development of autoimmune disease. J Immunol 171:6838–6845.

Christen U, Von Herrath MG. 2002. Apoptosis of autoreactive CD8 lymphocytes as a potential mechanism for the abrogation of type 1 diabetes by islet-specific TNF-alpha expression at a time when the autoimmune process is already ongoing. Ann N Y Acad Sci 958:166–169.

Christen U, von Herrath MG. 2004a. Induction, acceleration or prevention of autoimmunity by molecular mimicry. Mol Immunol 40:1113–1120.

Christen U, von Herrath MG. 2004b. IP-10 and type 1 diabetes: A question of time and location. Autoimmunity 37:273–282.

Christen U, von Herrath MG. 2004c. Manipulating the Type 1 vs Type 2 balance in Type 1 diabetes. Immunol Res 30:309–326.

Christen U, von Herrath MG. 2005. Infections and autoimmunity–good or bad? J Immunol 174:7481–7486.

Christen U, Wolfe T, Mohrle U, Hughes AC, Rodrigo E, Green EA, Flavell RA, von Herrath MG. 2001. A dual role for TNF-alpha in type 1 diabetes: Islet-specific expression abrogates the ongoing autoimmune process when induced late but not early during pathogenesis. J Immunol 166:7023–7032.

Coon B, An L-L, Whitton JL, von Herrath MG. 1999. DNA immunization to prevent autoimmune diabetes. J Clin Invest 104:189–194.

Ding L, Linsley PS, Huang LY, Germain RN, Shevach EM. 1993. IL-10 inhibits macrophage costimulatory activity by selectively inhibiting the up-regulation of B7 expression. J Immunol 151:1224–1234.

Dockter J, Evans CF, Tishon A, Oldstone MB. 1996. Competitive selection *in vivo* by a cell for one variant over another: Implications for RNA virus quasispecies *in vivo*. J Virol 70:1799–1803.

Ejrnaes M, Filippi CM, Ling EM, Togher LM, Crotty S, von Herrath MG. 2006. Resolution of a chronic viral infection following IL-10 receptor blockade. Submmited manuscript for Nature Medicine. (Manuscript submitted)

Ejrnaes M, Videbaek N, Christen U, Cooke A, Michelsen BK, von Herrath M. 2005. Different diabetogenic potential of auto-aggressive CD8 + clones associated with IFN-gamma-inducible protein 10 (CXC chemokine ligand 10) production but not cytokine expression, cytolytic activity, or homing characteristics. J Immunol 174:2746–2755.

Evans CF, Horwitz MS, Hobbs MV, Oldstone MB. 1996. Viral infection of transgenic mice expressing a viral protein in oligodendrocytes leads to chronic central nervous system autoimmune disease. J Exp Med 184:2371–2384.

Fife BT, Kennedy KJ, Paniagua MC, Lukacs NW, Kunkel SL, Luster AD, Karpus WJ. 2001. CXCL10 (IFN-gamma-inducible protein-10) control of encephalitogenic CD4 + T cell accumulation in the central nervous system during experimental autoimmune encephalomyelitis. J Immunol 166:7617–7624.

Fiorentino DF, Zlotnik A, Mosmann TR, Howard M, O'Garra A. 1991. IL-10 inhibits cytokine production by activated macrophages. J Immunol 147:3815–3822.

Frigerio S, Junt T, Lu B, Gerard C, Zumsteg U, Hollander GA, Piali L. 2002. beta cells are responsible for CXCR3-mediated T-cell infiltration in insulitis. Nat Med 8:1414–1420.

Gattass CR, King LB, Luster AD, Ashwell JD. 1994. Constitutive expression of interferon gamma-inducible protein 10 in lymphoid organs and inducible expression in T cells and thymocytes. J Exp Med 179:1373–1378.

Granelli-Piperno A, Golebiowska A, Trumpfheller C, Siegal FP, Steinman RM. 2004. HIV-1-infected monocyte-derived dendritic cells do not undergo maturation but can elicit IL-10 production and T cell regulation. Proc Natl Acad Sci U S A 101:7669–7674.

Hoffman LM, Fife BT, Begolka WS, Miller SD, Karpus WJ. 1999. Central nervous system chemokine expression during Theiler's virus-induced demyelinating disease. J Neurovirol 5:635–642.

Homann D, Dyrberg T, Petersen J, Oldstone MBA, von Herrath MG. 1999. Insulin in oral immune "tolerance": A one-amino acid change in the B chain makes the difference. J Immunol 163:1833–1838.

Homann D, Teyton L, Oldstone MB. 2001. Differential regulation of antiviral T-cell immunity results in stable CD8 + but declining CD4 + T-cell memory. Nat Med 7:913–919.

Jamieson BD, Butler LD, Ahmed R. 1987. Effective clearance of a persistent viral infection requires cooperation between virus-specific Lyt2 + T cells and nonspecific bone marrow-derived cells. J Virol 61:3930–3937.

Ji J, Sahu GK, Braciale VL, Cloyd MW. 2005. HIV-1 induces IL-10 production in human monocytes via a CD4-independent pathway. Int Immunol 17:729–736.

Jinquan T, Larsen CG, Gesser B, Matsushima K, Thestrup-Pedersen K. 1993. Human IL-10 is a chemoattractant for CD8 + T lymphocytes and an inhibitor of IL-8-induced CD4 + T lymphocyte migration. J Immunol 151:4545–4551.

Kaech SM, Hemby S, Kersh E, Ahmed R. 2002. Molecular and functional profiling of memory CD8 T cell differentiation. Cell 111:837–851.

Kamar N, Boulestin A, Selves J, Esposito L, Sandres-Saune K, Stebenet M, Chatelut E, Durand D, Rostaing L, Izopet J. 2005. Factors accelerating liver fibrosis progression in renal transplant patients receiving ribavirin monotherapy for chronic hepatitis C. J Med Virol 76:61–68.

Kasama T, Strieter RM, Lukacs NW, Burdick MD, Kunkel SL. 1994. Regulation of neutrophil-derived chemokine expression by IL-10. J Immunol 152:3559–3569.

Khan IA, MacLean JA, Lee FS, Casciotti L, DeHaan E, Schwartzman JD, Luster AD. 2000. IP-10 is critical for effector T cell trafficking and host survival in Toxoplasma gondii infection. Immunity 12:483–494.

Kolb SA, Sporer B, Lahrtz F, Koedel U, Pfister HW, Fontana A. 1999. Identification of a T cell chemotactic factor in the cerebrospinal fluid of HIV-1-infected individuals as interferon-gamma inducible protein 10. J Neuroimmunol 93:172–181.

Lane TE, Asensio VC, Yu N, Paoletti AD, Campbell IL, Buchmeier MJ. 1998. Dynamic regulation of alpha- and beta-chemokine expression in the central nervous system during mouse hepatitis virus-induced demyelinating disease. J Immunol 160:970–978.

Lau LL, Jamieson BD, Somasundaram T, Ahmed R. 1994. Cytotoxic T-cell memory without antigen. Nature 369:648–652.

Lin MY, Welsh RM. 1998. Stability and diversity of T cell receptor repertoire usage during lymphocytic choriomeningitis virus infection of mice. J Exp Med 188:1993–2005.

Liu MT, Chen BP, Oertel P, Buchmeier MJ, Armstrong D, Hamilton TA, Lane TE. 2000. The T cell chemoattractant IFN-inducible protein 10 is essential in host defense against viral-induced neurologic disease. J Immunol 165:2327–2330.

Liu YJ. 2001. Dendritic cell subsets and lineages, and their functions in innate and adaptive immunity. Cell 106:259–262.

Lo D, Freedman J, Hesse S, Palmiter RD, Brinster RL, Sherman LA. 1992. Peripheral tolerance to an islet cell-specific hemagglutinin transgene affects both CD4+ and CD8+ T cells. Eur J Immunol 22:1013–1022.

Lo D, Reilly CR, Scott B, Liblau R, McDevitt HO, Burkly LC. 1993. Antigen-presenting cells in adoptively transferred and spontaneous autoimmune diabetes. Eur J Immunol 23:1693–1698.

Loetscher M, Gerber B, Loetscher P, Jones SA, Piali L, Clark-Lewis I, Baggiolini M, Moser B. 1996. Chemokine receptor specific for IP10 and Mig: Structure, function, and expression in activated lymphocytes. J Exp Med 184:963–969.

Luster AD, Cardiff RD, MacLean JA, Crowe K, Granstein RD. 1998. Delayed wound healing and disorganized neovascularization in transgenic mice expressing the IP-10 chemokine. Proc Assoc Am Physicians 110:183–196.

Luster AD, Ravetch JV. 1987. Biochemical characterization of a gamma interferon-inducible cytokine (IP-10). J Exp Med 166:1084–1097.

Luster AD, Unkeless JC, Ravetch JV. 1985. Gamma-interferon transcriptionally regulates an early-response gene containing homology to platelet proteins. Nature 315:672–676.

Maldonado-Lopez R, De Smedt T, Michel P, Godfroid J, Pajak B, Heirman C, Thielemans K, Leo O, Urbain J, Moser M. 1999. CD8alpha + and CD8alpha- subclasses of dendritic cells direct the development of distinct T helper cells *in vivo.* J Exp Med 189:587–592.

Maldonado-Lopez R, Maliszewski C, Urbain J, Moser M. 2001. Cytokines regulate the capacity of CD8alpha(+) and CD8alpha(−) dendritic cells to prime Th1/Th2 cells *in vivo.* J Immunol 167:4345–4350.

Marker O, Volkert M. 1973. Studies on cell-mediated immunity to lymphocytic choriomeningitis virus in mice. J Exp Med 137:1511–1525.

Matloubian M, Somasundaram T, Kolhekar SR, Selvakumar R, Ahmed R. 1990. Genetic basis of viral persistence: Single amino acid change in the viral glycoprotein affects ability of lymphocytic choriomeningitis virus to persist in adult mice. J Exp Med 172:1043–1048.

McGavern DB, Christen U, Oldstone MB. 2002. Molecular anatomy of antigen-specific CD8(+) T cell engagement and synapse formation *in vivo.* Nat Immunol 3:918–925.

Miller SD, Katz-Levy Y, Neville KL, Vanderlugt CL. 2001. Virus-induced autoimmunity: Epitope spreading to myelin auto-epitopes in Theiler's virus infection of the central nervous system. Adv Virus Res 56:199–217.

Mocellin S, Wang E, Marincola FM. 2001. Cytokines and immune response in the tumor microenvironment. J Immunother 24:392–407.

Moore KW, de Waal Malefyt R, Coffman RL, O'Garra A. 2001. Interleukin-10 and the interleukin-10 receptor. Annu Rev Immunol 19:683–765.

Morimoto J, Yoneyama H, Shimada A, Shigihara T, Yamada S, Oikawa Y, Matsushima K, Saruta T, Narumi S. 2004. CXC chemokine ligand 10 neutralization suppresses the occurrence of diabetes in nonobese diabetic mice through enhanced beta cell proliferation without affecting insulitis. J Immunol 173:7017–7024.

Moskophidis D, Assmann-Wischer U, Simon MM, Lehmann-Grube F. 1987. The immune response of the mouse to lymphocytic choriomeningitis virus. V. High numbers of cytolytic T lymphocytes are generated in the spleen during acute infection. Eur J Immunol 17:937–942.

Moskophidis D, Lechner F, Pircher H, Zinkernagel RM. 1993. Virus persistence in acutely infected immunocompetent mice by exhaustion of antiviral cytotoxic effector T cells. Nature 362:758–761.

Mosmann TR, Coffman RL. 1989. Th1 and Th2 cells: Different patterns of lymphokine secretion lead to different functional properties. Annu Rev Immunol 7:145–173.

Murali-Krishna K, Altman JD, Suresh M, Sourdive DJ, Zajac AJ, Miller JD, Slansky J, Ahmed R. 1998. Counting antigen-specific CD8 T cells: A reevaluation of bystander activation during viral infection. Immunity 8:177–187.

Murali-Krishna K, Lau LL, Sambhara S, Lemonnier F, Altman J, Ahmed R. 1999. Persistence of memory CD8 T cells in MHC class I-deficient mice. Science 286:1377–1381.

Ohashi P, Oehen S, Buerki K, Pircher H, Ohashi C, Odermatt B, Malissen B, Zinkernagel R, Hengartner H. 1991. Ablation of tolerance and induction of diabetes by virus infection in viral antigen transgenic mice. Cell 65:305–317.

Oldstone MB. 1988. Prevention of type I diabetes in nonobese diabetic mice by virus infection. Science 239:500–502.

Oldstone MB. 1990. Viruses as therapeutic agents. I. Treatment of nonobese insulin- dependent diabetes mice with virus prevents insulin-dependent diabetes mellitus while maintaining general immune competence. J Exp Med 171:2077–2089.

Oldstone MB, Blount P, Southern PJ, Lampert PW. 1986. Cytoimmunotherapy for persistent virus infection reveals a unique clearance pattern from the central nervous system. Nature 321:239–243.

Oldstone MBA. 1989. Molecular mimicry as a mechanism for the cause and as a probe uncovering etiologic agent(s) of autoimmune disease. Curr. Top. Microbiol. Immunol. 145:127–136.

Oldstone MBA, Nerenberg M, Southern P, Price J, Lewicki H. 1991. Virus infection triggers insulin-dependent diabetes mellitus in a transgenic model: Role of anti-self (virus) immune response. Cell 65:319–331.

Olson JK, Croxford JL, Calenoff MA, Dal Canto MC, Miller SD. 2001. A virus-induced molecular mimicry model of multiple sclerosis. J Clin Invest 108:311–318.

Palella Jr., FJ, Delaney KM, Moorman AC, Loveless MO, Fuhrer J, Satten GA, Aschman DJ, Holmberg SD. 1998. Declining morbidity and mortality among patients with advanced human immunodeficiency virus infection. HIV Outpatient Study Investigators. N Engl J Med 338:853–860.

Pestka S, Krause CD, Sarkar D, Walter MR, Shi Y, Fisher PB. 2004. Interleukin-10 and related cytokines and receptors. Annu Rev Immunol 22:929–979.

Re F, Strominger JL. 2004. IL-10 released by concomitant TLR2 stimulation blocks the induction of a subset of Th1 cytokines that are specifically induced by TLR4 or TLR3 in human dendritic cells. J Immunol 173:7548–7555.

Rhode A, Pauza ME, Barral AM, Rodrigo E, Oldstone MB, von Herrath MG, Christen U. 2005. Islet-specific expression of CXCL10 causes spontaneous islet infiltration and accelerates diabetes development. J Immunol 175:3516–3524.

Rigopoulou EI, Abbott WG, Haigh P, Naoumov NV. 2005. Blocking of interleukin-10 receptor—a novel approach to stimulate T-helper cell type 1 responses to hepatitis C virus. Clin Immunol 117:57–64.

Sallusto F, Lanzavecchia A, Mackay CR. 1998. Chemokines and chemokine receptors in T-cell priming and Th1/Th2-mediated responses. Immunol Today 19:568–574.

Salvato M, Borrow P, Shimomaye E, Oldstone MB. 1991. Molecular basis of viral persistence: A single amino acid change in the glycoprotein of lymphocytic choriomeningitis virus is associated with suppression of the antiviral cytotoxic T-lymphocyte response and establishment of persistence. J Virol 65:1863–1869.

Seewaldt S, Thomas H, Ejrnaes M, Christen U, Wolfe T, Rodrigo E, Coon B, Michelsen B, Kay T, von Herrath MG. 2000. Virus-induced autoimune diabetes: Most b-cells die through inflammatory cytokines and not perforin from autoreactive (anti-viral) CTL. Diabetes 49:1801–1809.

Sevilla N, McGavern DB, Teng C, Kunz S, Oldstone MB. 2004. Viral targeting of hematopoietic progenitors and inhibition of DC maturation as a dual strategy for immune subversion. J Clin Invest 113:737–745.

Smelt SC, Borrow P, Kunz S, Cao W, Tishon A, Lewicki H, Campbell KP, Oldstone MB. 2001. Differences in affinity of binding of lymphocytic choriomeningitis virus strains to the cellular receptor alpha-dystroglycan correlate with viral tropism and disease kinetics. J Virol 75:448–457.

Sozzani S, Ghezzi S, Iannolo G, Luini W, Borsatti A, Polentarutti N, Sica A, Locati M, Mackay C, Wells TN, Biswas P, Vicenzi E, Poli G, Mantovani A. 1998. Interleukin 10 increases CCR5 expression and HIV infection in human monocytes. J Exp Med 187:439–444.

Takayama T, Morelli AE, Onai N, Hirao M, Matsushima K, Tahara H, Thomson AW. 2001. Mammalian and viral IL-10 enhance C-C chemokine receptor 5 but down-regulate C-C chemokine receptor 7 expression by myeloid dendritic cells: Impact on chemotactic responses and *in vivo* homing ability. J Immunol 166:7136–7143.

Torriani FJ, Rodriguez-Torres M, Rockstroh JK, Lissen E, Gonzalez-Garcia J, Lazzarin A, Carosi G, Sasadeusz J, Katlama C, Montaner J, Sette Jr., H, Passe S, De Pamphilis J, Duff F, Schrenk UM, Dieterich DT. 2004. Peginterferon Alfa-2a plus ribavirin for chronic hepatitis C virus infection in HIV-infected patients. N Engl J Med 351:438–450.

Vicari AP, Chiodoni C, Vaure C, Ait-Yahia S, Dercamp C, Matsos F, Reynard O, Taverne C, Merle P, Colombo MP, O'Garra A,

Trinchieri G, Caux C. 2002. Reversal of tumor-induced dendritic cell paralysis by CpG immunostimulatory oligonucleotide and anti-interleukin 10 receptor antibody. J Exp Med 196:541–549.

Vicari AP, Trinchieri G. 2004. Interleukin-10 in viral diseases and cancer: Exiting the labyrinth? Immunol Rev 202:223–236.

von Herrath MG, Dockter J, Oldstone MBA. 1994. How virus induces a rapid or slow onset insulin-dependent diabetes mellitus in a transgenic model. Immunity 1:231–242.

Willems F, Marchant A, Delville JP, Gerard C, Delvaux A, Velu T, de Boer M, Goldman M. 1994. Interleukin-10 inhibits B7 and intercellular adhesion molecule-1 expression on human monocytes. Eur J Immunol 24:1007–1009.

Wolk K, Kunz S, Asadullah K, Sabat R. 2002. Cutting edge: Immune cells as sources and targets of the IL-10 family members? J Immunol 168:5397–5402.

Zinkernagel RM, Doherty PC. 1974. Restriction of in vitro T cell-mediated cytotoxicity in lymphocytic choriomeningitis within a syngeneic or semiallogeneic system. Nature 248: 701–702.

Zinkernagel RM, Welsh RM. 1976. H-2 compatibility requirement for virus-specific T cell-mediated effector functions *in vivo*. I. Specificity of T cells conferring antiviral protection against lymphocytic choriomeningitis virus is associated with H-2K and H-2D. J Immunol 117:1495–1502.

